# Cystic Duct Remnant–Duodenal Fistula following Laparoscopic Cholecystectomy: A Case Report and Literature Review

**DOI:** 10.3390/reports7010016

**Published:** 2024-02-23

**Authors:** Aleksandra Polikarpova, Ngee-Soon Lau, David Yeo

**Affiliations:** 1 Department of Upper Gastrointestinal and Hepatobiliary Surgery, Royal Prince Alfred Hospital, Sydney, NSW 2050, Australiadyeo@med.usyd.edu.au (D.Y.); 2 Institute of Academic Surgery, Royal Prince Alfred Hospital, Camperdown, Sydney, NSW 2050, Australia; 3 Australian National Liver Transplantation Unit, Royal Prince Alfred Hospital, Sydney, NSW 2050, Australia; 4 Faculty of Medicine and Health, University of Sydney, Sydney, NSW 2050, Australia; 5 Surgical Outcomes Research Centre (SOuRCe), Royal Prince Alfred Hospital, Sydney, NSW 2050, Australia

**Keywords:** laparoscopic cholecystectomy, biliary fistula, digestive system fistula, enterobiliary fistula, ERCP

## Abstract

Laparoscopic cholecystectomy is the most common procedure performed for the management of symptomatic gallstone disease. This, however, can be complicated by the formation of fistulous communications between the biliary tree and the gastrointestinal tract. This abnormal communication allows for the flow of bile and bowel contents between two systems (biliary system and intestine), which can cause abdominal pain, nausea, vomiting, and biliary sepsis. We would like to present a rare case of fistulous communication between the cystic duct stump and duodenum and outline possible contributing factors. The literature review describes the most common interventions for the management of fistulas with emphasis on ERCP and stent preferences to eliminate transpapillary pressure gradient, which directly contributes to fistula closure.

## 1. Introduction

Enterobiliary fistulas are abnormal connections that form between the biliary system (gallbladder, and bile ducts) and the intestines. These fistulas allow for the passage of bile and intestinal contents between these two systems, leading to various clinical symptoms and complications.

The biliary system plays a crucial role in the digestion and absorption of fats. Bile, produced by the liver and stored in the gallbladder, helps in the emulsification and breakdown of dietary fats, allowing them to be properly digested and absorbed in the intestines. Normally, bile flows from the liver through the bile ducts into the gallbladder, where it is concentrated and stored until it is required for digestion.

However, when an enterobiliary fistula forms, it disrupts the normal flow of the bile and intestinal contents. This can occur due to various underlying conditions, such as gallstones, trauma, infection, tumours, or complications following cholecystectomy or other surgical procedures.

The formation of an enterobiliary fistula can lead to a range of symptoms, including abdominal pain, jaundice, nausea, vomiting, and biliary sepsis. These symptoms can vary depending on the size and location of the fistula and the extent of bile and intestinal fluid leakage.

Enterobiliary fistulas can be diagnosed through various imaging studies, such as ultrasound, computed tomography (CT) scanning, magnetic resonance imaging (MRI), or endoscopic retrograde cholangiopancreatography (ERCP). Once diagnosed, the management of enterobiliary fistulas typically requires a multidisciplinary approach, involving surgeons, gastroenterologists, and radiologists.

Treatment options for enterobiliary fistulas depend on several factors, including the underlying cause, the size and location of the fistula, and the patient’s overall health. Presently, most biliary fistulas are treated with different endoscopic techniques during ERCP. However, surgical intervention is occasionally required to repair the fistula and restore the normal flow of bile and intestinal contents.

## 2. Detailed Case Description

A 70-year-old previously healthy man presented to the Emergency Department with worsening right upper quadrant abdominal pain that had been present since having an elective laparoscopic cholecystectomy for recurrent biliary colic 3 months prior. However, the details of the initial pre-operative assessment could not be retrieved as the original surgery was performed at a different hospital. An outpatient computed tomography (CT) scan revealed a collection measuring 14.4 × 4.9 × 6.0 cm adjacent to segment 6 of the liver. The collection had mildly thickened walls and contained multiple radiodensities suspicious for retained gallstones. There were multiple gas locules in the gallbladder fossa and extensive pneumobilia.

On arrival at the Emergency Department, he was hemodynamically stable but had marked tenderness in the right upper quadrant without features of peritonitis. His blood test revealed mild leukocytosis of 15.8 × 10^9^/L with elevated neutrophils to 13.8 × 10^9^/L, his haemoglobin was 117 g/L, and his C reactive protein was 170.8 mg/L. His liver function tests were unremarkable with Bilirubin 7 µmol/L, ALP 73 U/L, GGT 32 U/L, ALT 12 U/L, and AST 16 U/L; the lipase was 47 U/L.

His past medical history was remarkable for type II diabetes mellitus managed with oral hypoglycaemic agents, gastro-esophageal reflux disease, hypertension, and cholelithiasis. Surgical history was significant for laparoscopic cholecystectomy 3 months prior.

Given outpatient CT findings, the differentials to be considered were: (1) postoperative abscess with retained infected stones; (2) postoperative bile leak from the cystic stump and/or the duct of Luschka; (3) pneumobilia post- endoscopic retrograde cholangiopancreatography (ERCP); (4) pneumobilia due to bilio-duodenal/colonic fistula; and (5) missed malignancy.

The operative notes were reviewed, and the findings were as follows: thick gallbladder with multiple small stones, and frozen Callot’s triangle. A complete cholecystectomy was performed with no intraoperative cholangiography attempted. To differentiate further, the patient underwent a CT cholangiogram.

**Figure 1:**
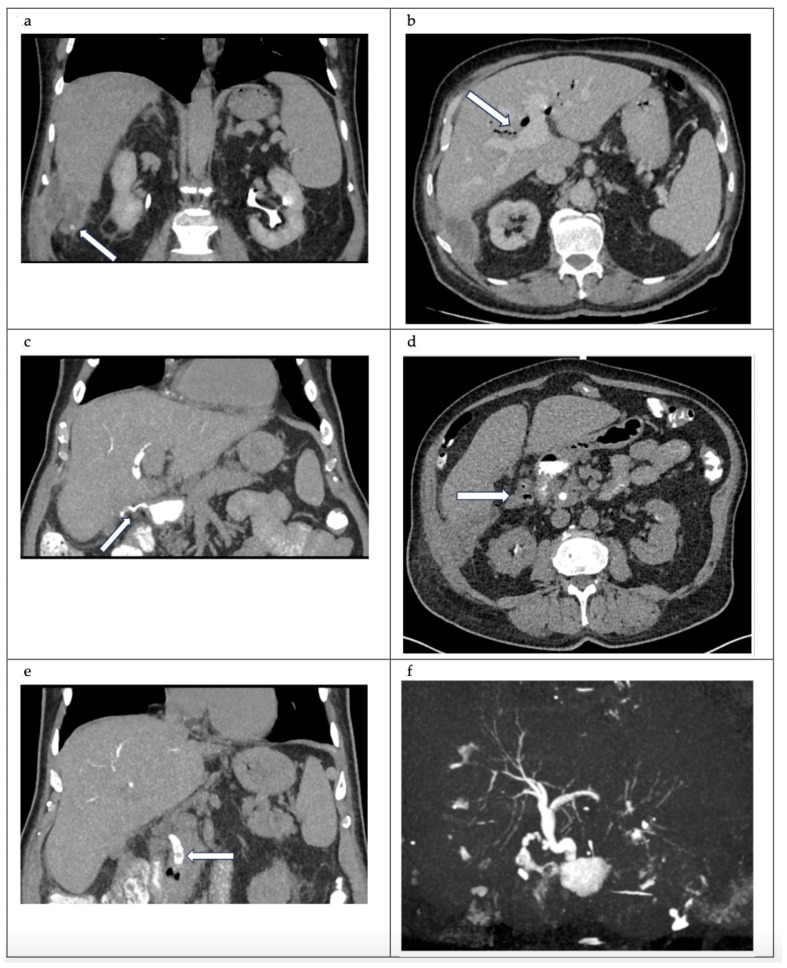
(**a**) Coronal view CT abdomen. Porto-venous phase. Adjacent to segment 6 of the liver, there is a 14.4 × 4.9 × 6 cm collection (white arrow). The collection has a mildly thickened wall. The collection contains multiple dependent calculi. (**b**) Axial view CT abdomen. Porto-venous phase. Marked pneumobilia (white arrow). (**c**) Coronal view CT cholangiogram. Contrast leak from the cystic stump into duodenum (white arrow). (**d**) Axial view CT cholangiogram—post-biliscopin phase. A lobulated and gas-containing outpouching immediately superior to the cholecystectomy clip, that communicated with the cystic duct, as well as a fistulous tract with the first part of the duodenum—representative of a fistula (white arrow outlines the collection). (**e**) Coronal view CT cholangiogram. 5 mm filling defect at the distal common bile duct (CBD) (white arrow) (**f**) 3D reconstructed model.

**Figure 2:**
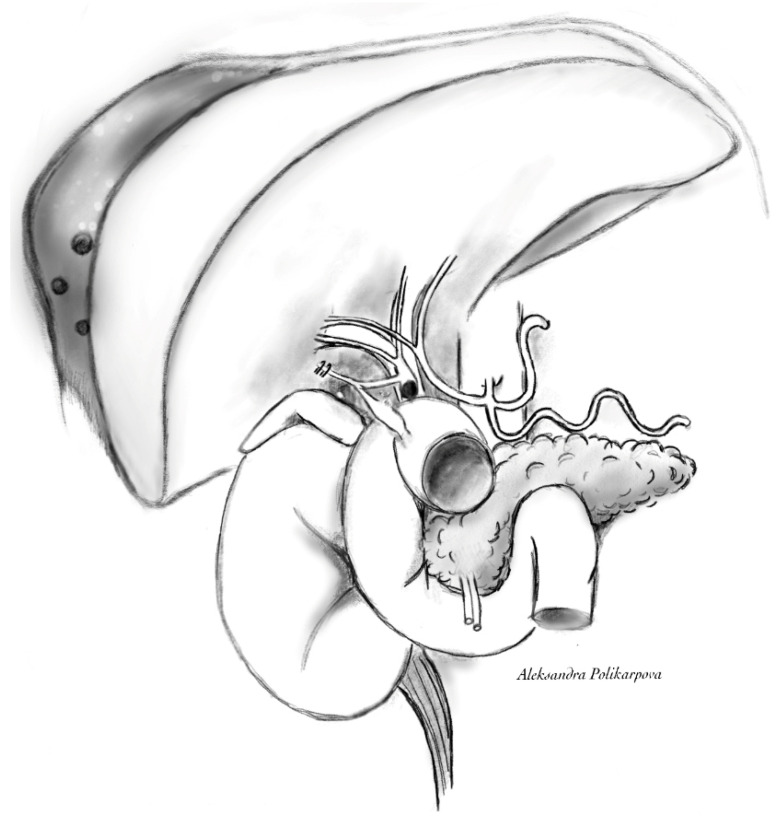
Cystic duct remnant—duodenal fistula, retained CBD stone and collection adjacent to segments 6 and 7. Schematic drawing.

An MRCP would be an alternative to a CT cholangiogram., in our case, however, MRCP was not available on short notice. The parahepatic collection and extensive pneumobilia were once again noted (Figure 1a,b). Four filling defects in the common bile duct were discovered, with the largest measuring 5 mm (Figure 1d). A gas-containing collection inferior to the cystic duct clips was identified with biliscopin extravasation into the collection and duodenum, which is representative of cystic duct remnant–duodenal fistula (Figure 1c,d). A 3D reconstruction image helps to visualise the anatomy of the biliary system (Figure 1f and Figure 2).

**Figure 3:**
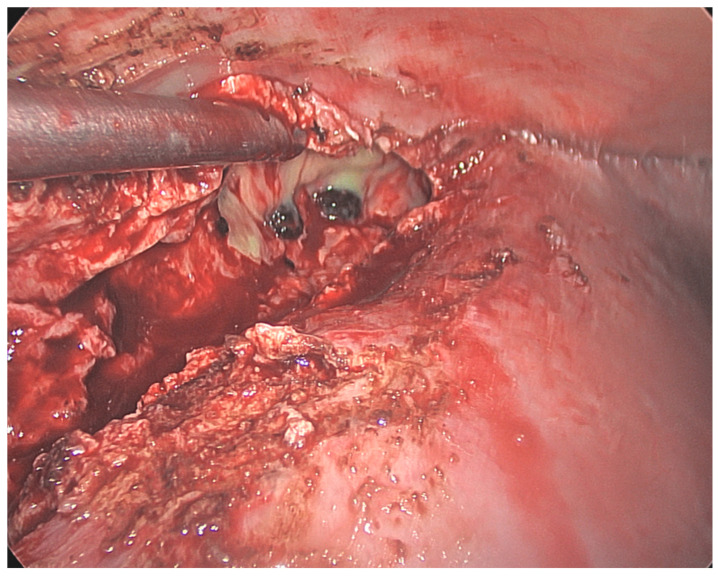
Intraoperative findings during laparoscopic washout. Suction instrument in the abscess cavity, groped gallstones evident in the cavity.

Given the findings of the stones in the common bile duct, fistulous communication with the first part of the duodenum, and a subhepatic multiloculated collection, the decision was to proceed with an ERCP and a laparoscopic washout of subhepatic collection. The patient was positioned supine, pneumoperitoneum was achieved via optical entry through reverse Palmer’s point, and two working ports in the epigastrium and right upper quadrant were placed. The gallbladder fossa was not dissected due to the presence of a fistula. The liver was mobilised medially to facilitate access to the collection. The collection was located near segments 6–7 and extended into the lateral abdominal wall. Several small, pigmented stones were identified and evacuated (Figure 3). The drain was left adjacent to segments 6–7. The ERCP revealed a suspected fistula opening in the first part of the duodenum and five filling defects within the common bile duct. Multiple stones were extracted, and a 7 Fr 7 cm pigtail stent was placed. The patient was discharged on postoperative day 5 following drain removal and the normalisation of inflammatory markers. The stent was removed six weeks later, the repeated ERCP demonstrated the resolution of the fistula.

## 3. Discussion

A biliary fistula is an abnormal connection between the biliary tree and another epithelial surface. It can involve the abdominal wall (external fistula) or gastrointestinal tract (internal fistula). Gallstones are the most common cause of biliary fistulas, accounting for 90% of enterobiliary fistulas [1]. Extrabiliary causes like peptic ulcer disease, malignancy, Crohn’s disease of the bowel, and trauma account for the other 10% [2]. The most common risk factors are female sex, recurrent cholangitis, and age over 74 [3]. Primary biliary fistulas are a direct complication of gallstone disease, while secondary fistulas can be caused by retained gallstones or injury during cholecystectomy. Biliary fistulas were initially described by Danish physician Thomas Bartholin in 1654 [4]. Two hundred years later, Ludwid Courvoisier published the first case series of 131 cases of gallstone ileus with a mortality rate of just over 40% [4]. In 1948, Pablo Luis Mirizzi reported a case of a gallstone impacted in the neck of the gallbladder that caused the compression of the hepatic duct [5]. This condition would be later named after him. In early classifications, only four types of Mirizzi syndrome were recognised [6]. However, it was recognised that fistulas from the gallbladder to the common bile duct or the hepatic duct are evolving stages of the same disease. Therefore, in 2008, Beltran proposed the inclusion of cholecystoenteric fistulas in the Mirizzi syndrome classification as type 5a (fistulas without gallstone ileus) and type 5b (fistulas with gallstone ileus). Beltran et al. described the “natural history of Mirizzi syndrome”, outlining that all classes of Mirizzi syndrome represent natural progression of the disease with biliary fistula formation as a late complication. The incidence of primary biliary fistulas remains low, with approximately 0.50–0.86% of patients developing fistulous communication [7]. Cholecystoduodenal fistulas are the most common, followed by cholecystogastric and cholecystocolic with prevalence rates of 40%, 32%, and 28%, respectively.

The incidence of secondary fistulas is also relatively low, at 0.3–0.6% [6]. The true incidence of cystic duct remnant–-duodenal fistulas is unknown. To our knowledge, only 10 case reports have been published describing fistulas between the cystic duct remnant and the stomach or bowel. Tsardakas, Van, and Nelson described cases similar to ours when fistulous communication was diagnosed following cholecystectomy [2,8,9]. Nakshabendi described a similar case of cystic duct remnant fistula. They, however, opted for ERCP without stent placement and a short course of antibiotics [10]. Sharma reported the appearance of a primary cystic duct–duodenal fistula, which was resected during open cholecystectomy [11]. Rogy et al. described a case associated with a long (>1.5 cm) cystic stump; no intervention was offered to the patient [12]. This is, perhaps, due to the inferior ERCP technique given the fact that the report was published three decades ago. A case report by Carotenuto described a patient with carcinomatous papilloma resulting in fistula formation [13].

The diagnosis of biliary fistulas remains challenging, with a preoperative detection rate of only 8–14%, and transabdominal ultrasound and CT remain the most common imaging modalities in the primary settings [6]. CT scans provide highly detailed images of the biliary anatomy, but they are only suitable for patients who have a serum bilirubin level of less than 3 mL dL^−1^. This is because the contrast used in CT scans binds to bilirubin, and if the contrast is held up due to slow transit, the images may not be representative.

The ultrasound features of biliary fistulas often include pneumobilia and the segmental thinning of the bladder wall, while CT scans may also show dropped gallstones and fistula tracts [14,15]. However, some of these features are not specific, as pneumobilia commonly arises from infection, emphysematous cholecystitis, biliary necrosis, pneumobilia post-ERCP, or Sphincter of Oddi dysfunction. Other limitations of ultrasound are operator dependency, body habitus, and the presence of intestinal air. Magnetic resonance cholangiopancreatography (MRCP) is a non-invasive alternative that is commonly used to diagnose biliary diseases, especially in patients with dilated bile ducts. Magnetic resonance cholangiopancreatography is another imaging modality used for patients where for whom the CT cholangiogram is contraindicated or failed to identify fistulous communication [15]. It is more accurate in defining the anatomy of the biliary tree and gallbladder and can detect fistulas in 75% of cases [16]. In our case, MRCP was not available in a timely manner; therefore, CT cholangiography was performed.

The management of primary biliary fistulas is still challenging. Precise understanding of biliary anatomy is pivotal as inflammation in Calot’s triangle can significantly alter the anatomy of the hilum, increasing the risk of bile duct injury during surgery.

Laparoscopic surgery is a viable option for treating primary fistulas associated with different types of Mirizzi syndrome. While in type I Mirizzi syndrome and biliary ileus, retrograde cholecystectomy is the traditional approach, anterograde surgery may be necessary for more complex cases. Katsohis et al. suggested subtotal cholecystectomy as an alternative for these patients [17]. If an exploration of the common bile duct is required, it is advisable to make a separate incision that can also serve as a drainage site for a T tube. In cases of type 2 Mirizzi syndrome, where the biliary tract is partially involved, the recommended surgery involves a subtotal cholecystectomy, leaving a small portion of the gallbladder wall (5 mm in size) for bile duct reconstruction. To protect the reconstructed area, the drainage of the bile duct by the T tube is performed. Lee et al. described combed laparoscopic and robotic approaches in a small number of cases [18].

In Mirizzi type 3 fistulas, the initial operative plan should be subtotal cholecystectomy with choledocoplasty. Hepaticojejunostomy is reserved for patients with large defects, as in type 4 fistulas [6,19].

The management of type 5 Mirrizi syndrome should focus on the resolution of gallstone ileus, followed by the delayed management of the cholecytoenteric fistulas [20].

Endoscopic retrograde cholangiopancreatography (ERCP) remains the gold standard technique for the management of secondary biliary fistulas. It provides adequate visualisation of the fistula and has diagnostic accuracy ranging from 55% to 90%, which is heavily operator- dependent. The primary goal of endoscopic fistula treatment is to equalise the pressure between the biliary tree and duodenum, allowing the defect to close spontaneously [21]. Given that distal obstruction from a stone, stricture, or stenosis of the papilla can increase intraductal pressure and promote and maintain the biliary fistula, the preferred methods are sphincterotomy, stenting, and nasobiliary tube placement alone, or any combination of the above [3,21,22,23,24,25]. Several observational uncontrolled observational studies in patients treated with stenting and sphincterotomy suggest that sphincterotomy can be avoided in patients with otherwise unobstructed ducts [23,24].

Foutch et al. stated that 7 Fr stents have a 22% failure rate, resulting in persistent bile leaks; these defects seal spontaneously with stent upsizing to 10 Fr [24]. In the majority of cases, the stent was inserted with the proximal end above the site of the fistula. There were no statistically significant outcomes reported between long (>7 cm) and short (<=3 cm) stents according to Sandha et al. [21]. However, Bjorkman et al. reported that short 2– 3 cm 10 Fr stents placed distal to the defect resulted in 100% leak resolution, which highlights the importance of the elimination of the transpapillary pressure gradient [25]. Operative management is reserved for patients requiring laparoscopic or open washout of collections. Approximately 10% of patients have persistent biliary defects despite stent placement with or without sphincterotomy. In such instances, the temporary use of a covered, self-expanding metal stent may provide a solution [26]. In the case of refractory bile leaks, one must consider that the origin of the leak is an abnormal take of the cystic duct which may arise from an anomalous aberrant right hepatic duct. These leaks frequently require surgical intervention such as hepaticojejunostomy [27].

## 4. Conclusions

Biliary fistulas are uncommon under-recognised pathologies that can present after the gallbladder has been removed. It is difficult to establish whether the patient described in this case report had a primary or secondary biliary fistula due to their complex pathology. Although the initial operation report did not identify a fistula, it stated dense adhesions, which could indicate the presence of a fistula. Since a cholangiogram was not performed, we have no means to confirm or exclude the presence of a primary fistula between the cystic duct and the duodenum. Alternatively, an unrecognised significant distal obstruction by a stone could be followed by a blowout of the cystic duct stump, resulting in bile leak and a secondary biliary fistula. This seems the most probable case given that the patient’s symptoms presented months after the initial surgery, as well as the findings of the CT cholangiogram. Two cases of cholecystocolic fistulas were reported by Rastogi and Ha, which were caused by gallbladder adenocarcinoma. Therefore, malignancy had to be considered [28,29]. The histopathology report from the initial cholecystectomy showed no malignant transformation.

In conclusion, this case is particularly interesting as it combines several uncommon complications of gallstone disease. The patient had a parahepatic pus collection, which perhaps was the reason for abdominal discomfort, a cystic duct remnant–duodenal fistula, and an obstructing calculus in the distal CBD. This once again highlights the paramount importance of understanding biliary anatomy. Infected gallstones and an ongoing inflammatory process leading to an increased transpapillary pressure are leading causes of biliary fistula formation. This case highlights the importance of biliary tree decompression in the management of biliary fistulas. ERCP is a safe and effective procedure for the diagnosis and management of biliary fistulas. Preference should be given to the larger calibre of stents to alleviate the pressure. Surgical treatment is reserved for defects refractory to endoscopic treatment.

## Data Availability

Data are contained within the article.
